# Sub-Zero Non-Freezing of Vascularized Composite Allografts Preservation in Rodents

**DOI:** 10.21203/rs.3.rs-3750450/v1

**Published:** 2023-12-25

**Authors:** Irina Filz von Reiterdank, Pierre Tawa, Yanis Berkane, Eloi de Clermont-Tonnerre, Antonia Dinicu, Casie Pendexter, Marion Goutard, Alexandre G. Lellouch, Aebele B Mink van der Molen, J. Henk Coert, Curtis L. Cetrulo, Korkut Uygun

**Affiliations:** Center for Engineering in Medicine and Surgery, Derpartment of Surgery, Massachusetts General Hospital, Harvard Medical School; Department of Plastic, Reconstructive and Aesthetic Surgery, Hôpital Paris Saint-Joseph; Department of Plastic, Reconstructive and Aesthetic Surgery, Hôpital Sud, CHU Rennes, University of Rennes; Department of Plastic, Reconstructive and Aesthetic Surgery, Hôpital Paris Saint-Joseph; Center for Engineering in Medicine and Surgery, Derpartment of Surgery, Massachusetts General Hospital, Harvard Medical School; Center for Engineering in Medicine and Surgery, Derpartment of Surgery, Massachusetts General Hospital, Harvard Medical School; Department of Plastic, Reconstructive and Aesthetic Surgery, Hôpital Paris Saint-Joseph; Innovative Therapies in Haemostasis, INSERM UMR-S 1140, University of Paris, F-75006; Department of Plastic, Reconstructive and Hand Surgery, University Medical Center Utrecht, Utrecht University; Department of Plastic, Reconstructive and Hand Surgery, University Medical Center Utrecht, Utrecht University; Vascularized Composite Allotransplantation Laboratory, Massachusetts General Hospital, Harvard Medical School; Center for Engineering in Medicine and Surgery, Derpartment of Surgery, Massachusetts General Hospital, Harvard Medical School

## Abstract

Ischemia is a major limiting factor in Vascularized Composite Allotransplantation (VCA) as irreversible muscular injury can occur after as early as 4–6 hours of static cold storage (SCS). Organ preservation technologies have led to the development of storage protocols extending rat liver ex vivo preservation up to 4 days. Development of such a protocol for VCAs has the added challenge of inherent ice nucleating factors of the graft, therefore this study focused on developing a robust protocol for VCA supercooling. Rodent partial hindlimbs underwent subnormothermic machine perfusion (SNMP) with several loading solutions, followed by cryoprotective agent (CPA) cocktail developed for VCAs. Storage occurred in suspended animation for 24h and VCAs were recovered using SNMP with modified Steen. This study shows a robust VCA supercooling preservation protocol in a rodent model. Further optimization is expected to allow for its application in a transplantation model, which would be a breakthrough in the field of VCA preservation.

## INTRODUCTION

Vascularized Composite Allotransplantation (VCA) has rapidly emerged as a valuable treatment for patients with amputations over the past few decades [1]. Similar to solid organ transplantation (SOT), reducing ischemia time is a major concern in VCA as it directly influences graft viability and functional outcomes [2, 3]. The current gold standard for organ preservation is static cold storage (SCS), which involves the use of preservative agents and maintaining low temperatures around 4°C [4]. However, in VCA, irreversible muscle injury can already occur after 4–6 hours of SCS, limiting the potential for functional rehabilitation and increasing the risk of rejection episodes [5, 6].

Storage limitation has direct effects on auto-transplantation and indirect effects on allotransplantation. Clinically, patients require reconstruction of severed extremities or parts thereof within 6h, including logistical challenges such as transportation and surgical planning. For traumatic amputations this means replantation is often not possible due to remoteness of patients or instability of vital functions requiring prior treatment not allowing for immediate reconstructive surgery. In terms of allotransplantation, patient matching based on HLA, skin color, age, sex and size is necessary before transplantation can occur [7]. Even if these hurdles can be overcome, rejection of VCAs remain a major challenge as 89% of patients undergo an acute rejection episode and 11% a chronic rejection episode [8]. While VCAs have the potential to offer outcomes for severely disfigured patients for which few to no other options exist, the risks of these non-vital transplants need to be considered on a more balanced scale. The immunosuppressive aspect of transplantation is particularly crucial in VCA since transplants are performed to improve quality of life, rather than being life-saving. Moreover, patients are typically young and in good health, making them more susceptible to the long-term adverse effects of immunosuppression [8]. Additionally, these grafts contain skin tissue, which is known to be highly immunogenic [9]. Recent research focuses on developing tolerance induction protocols through mixed chimerism that aim to reduce the need for immunosuppressive treatments [10, 11]. However, for VCAs live donation is not an option [12] and tolerance protocols require 48h of patient preparation prior to transplantation (conditioning period), thereby making extended organ storage a priority. Especially preservation of functional muscle tissue is a major challenge here. Therefore, it is imperative to explore strategies that extend the preservation duration of allografts to address these aforementioned concerns. Preservation techniques enabling *organ banking* have been developed for liver, kidney, lung and heart models. However, a reliable storage protocol for VCAs allowing for extended storage is lacking [13].

Drawing inspiration from biomimicry [14], novel organ preservation technologies utilizing negative temperatures have been developed to prolong graft preservation [15]. Among these, Sub-Zero Non-Freezing (SZNF) protocols aim to achieve negative temperatures without ice nucleation, enabling slower metabolism while avoiding freezing injuries. In a previous study, our team successfully extended the preservation duration of rat livers up to four days while maintaining viability using a new supercooling protocol that combined cryoprotective agents (CPAs) with strict avoidance of ice nucleating factors [16–18]. In this study, the objective is to establish a robust SZNF preservation protocol tailored to and allowing for supercooling of VCAs and perform initial optimization with the aim of laying the groundwork for future application to *in vivo* models.

## METHODS

### Animals

Forty-three inbred, male Lewis rats (250 ± 50 grams) were used for all experiments (Charles River Laboratories, Wilmington, MA). The animals received humane care in accordance with the National Research Council guidelines and the experimental protocols were approved by the IACUC of Massachusetts General Hospital (Boston, MA) and the Animal Care and Use Review Office. Authors complied with the ARRIVE guidelines.

### Study design

**Figure 1:**
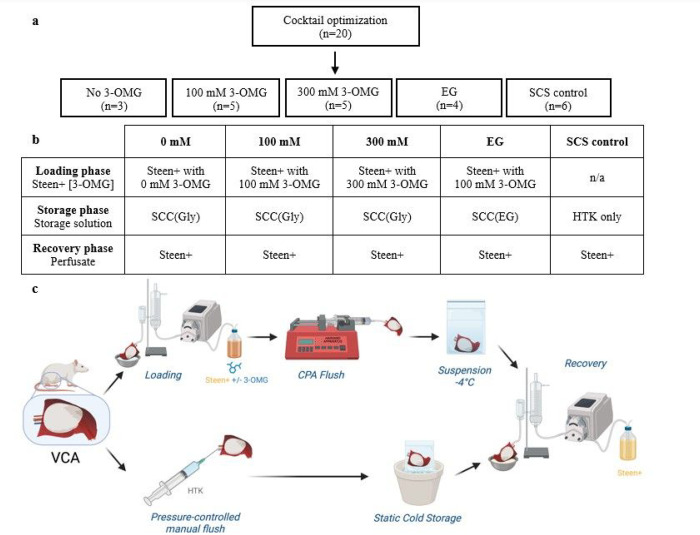
(**a**) Experimental design of the study. Each group consists of 3–6 animals. Twenty animals were used to complete phase 1, which allowed for development of a VCA SZNF protocol. (**b**) Solutions used in each phase are shown per group. Supercooling cocktail consists of 5g PEG + 50 mM trehalose + 5% glycerol (Gly) or 5% ethylene glycol (EG) diluted in HTK. (**c**) VCAs were subjected to either the supercooling protocol, or cold stored. *EG ethylene glycol; Gly glycerol; HTK Histidine-Tryptophan-Ketoglutarate; mM mili Molair; SCC Supercooling Cocktail; 3-OMG 3-O-methyl-d-glucose*.

Experimental groups and their solutions are displayed in Fig. 1a–b. Experiments were conducted in three phases: (1) determination of a CPA cocktail that allows reliable SZNF of rodent VCAs; (2) determination of optimal 3-O-Methyl-d-Glucose (3-OMG) concentration (300 mM vs 100 mM vs no 3-OMG); (3) evaluation of the perfusion parameters after 24h storage (SZNF vs SCS); (4) comparison of histological outcomes after SNMP recovery. As an alternative to glycerol in the CPA cocktail, ethylene glycol (EG) was chosen based on its lower viscosity and its positive effects on weight gain and viability in liver cryopreservation [19].

### VCA procurement

After induction using isoflurane (5%) inhalation with 100% O_2_, general anesthesia was sustained with inhaled isoflurane (1–3%) and anesthesia depth was confirmed with a toe pinch test. Partial hindlimbs were procured as previously described [20]. Briefly, grafts include the knee joint with 10 mm distal femur and 10 mm proximal femur and tibia, along with thigh muscle groups, the inguinal fat pad and calf muscles as well as the surrounding skin paddle. Femoral vessels were skeletonized and ligated 5 minutes after IV administration of 100IU/mL/kg heparin in the penile dorsal vein. Femoral artery was cannulated with a 24G angio catheter and secured with 6/0 nylon suture. Femoral vein was cut after ligation. Immediately after procurement, a pressure-controlled manual flush with 3 mL (200IU) of heparin saline at room temperature was performed. Next, the VCA was subjected to either SNMP to initiate loading phase, or cold stored as described below (Fig. 1c).

### Machine perfusion system

Perfusate was circulated using a roller pump system (Masterflex L/S, Vernon Hills, IL) with two separate sets of tubing (Masterflex platinum-cured silicone tubing, L/S 13, Cole-Parmer, Vernon Hills, IL) delivering perfusate into and out of the perfusion reservoir. Temperature was regulated by a water bath (Polystat Cooling/Heating Circulating Bath, Cole-Parmer), set at 21°C or 4°C depending on the phase, through double-jacketed perfusion system components (Radnoti, Covina, CA, USA). Perfusate oxygen concentration was maintained within a close range of 450 mmHg using a 95% O2/5% CO2 gas cylinder (Airgas, Radnor, PA, USA). Pressure transducer (PT-F, Living Systems Instrumentation, St Albans City, VT) was connected close to the angio catheter (BD Angiocath 24G) in the femoral artery during perfusion.

Vascular resistance (flow and pressure) was monitored continuously; flow rate was manually adjusted to reach a target pressure of 30–35 mmHg. Blood gas and electrolytes in the inflow and outflow perfusate were measured at 30, 60 and 90 minutes during the loading phase and at 60, 90 and 120 minutes during recovery phase using the i-STAT blood gas analysis machine (Abbott, Princeton, NJ). Oxygen consumption was calculated using a modified Fick equation using circuit flow, limb weight, and pre- and post-limb oxygen contents. Weight (g) was measured after procurement and at the end of each phase. Once the perfusate was warmed to 21°C and oxygenated, pO_2_, pCO_2_ and pH of the solution were verified on the i-STAT machine, and NaHCO_3_^−^ titration was performed to correct for acidosis if needed.

**Figure 2:**
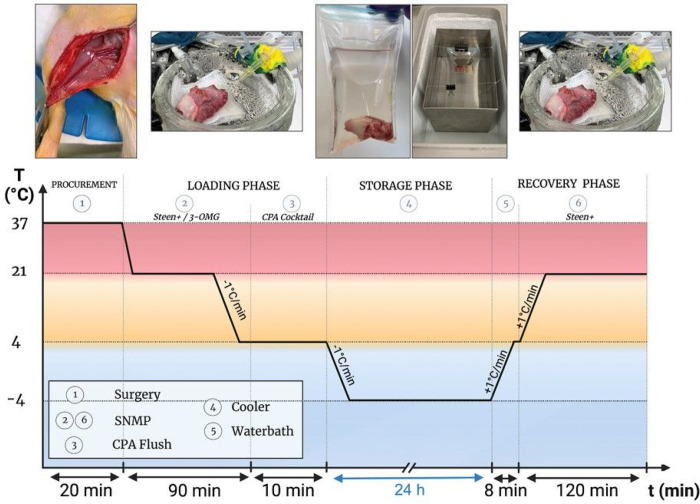
Supercooling protocol for rodents. SNMP with modified Steen with (100 mM vs 300 mM) or without 3-OMG is initiated shortly after procurement (WIT < 12 min.). After 60 minutes, temperature is decreased to 4°C until 90 minutes of perfusion is reached. VCAs are detached from the system and flushed with the CPA cocktail (HTK + 5g PEG + 50 mM trehalose + 5% glycerol) with a flow of 0.5 mL/min. After submersion in 80 mL of the CPA cocktail, bags are sealed to remove air-liquid interface and stored in refrigerant at -4°C for 24h. VCAs are rewarmed for 8 min in a water bath and recovered using SNMP with modified Steen for 2h. CPA cryoprotective agents; h hour(s); min minute(s); *SNMP subnormothermic machine perfusion; Steen+ modified Steen; 3-OMG 3-O-methyl-d-glucose*.

Machine perfusion protocol consists of three phases: (1) loading phase; (2) storage phase; (3) recovery phase, as described in Fig. 2.

### Loading phase

Based on successes in liver preservation [16], the first 60 minutes of the loading phase consisted of 3-O-methyl-glucose (3-OMG) loading for intracellular cryoprotection. At 60 minutes, temperature was lowered to 4°C until 90 minutes is reached. Next, VCA is detached from the system and flushed with 5 mL CPA cocktail (HTK + PEG + 50 mM trehalose + 5% glycerol) with a flow of 0.5 mL/min. In the optimization phase, a large range of cocktails with CPAs at different concentrations were tested to assess freezing points (data not shown). Based on these studies the cocktail above was chosen.

### Storage phase

Two storage methods were compared: (a) supercooling (SZNF) and (b) static cold storage (SCS) as shown in Fig. 1c. In the experimental groups VCAs were stored in sealed bags, removing the air-liquid interface, and submerged in refrigerant at −4°C to minimize ice nucleating factors such as vibrations. SCS control VCAs were manually flushed using pressure control between 40–60 mmHg with 5 mL of 4°C Histidine-Tryptophan-Ketoglutarate (HTK) solution. VCAs were weighed and submersed in a sterile bag containing 80 mL of 4°C HTK, after which they were stored at 4°C for 24h. After storage, VCAs were weighed and underwent the same recovery protocol as supercooled groups.

### Recovery phase

To avoid the occurrence of ice formation, VCAs were gradually rewarmed to 4°C in a water bath at 37°C for 8 minutes. Perfusion was initiated at 4°C with modified Steen [21]. Upon connection of the organ, temperature was increased to 21°C and continued for 2h.

### Histology

After recovery phase, 3×3 mm biopsies were taken of skin, muscle and vasculature. Biopsies were fixed in formalin and processed for histopathological examination. Slides were stained with hematoxylin and eosin (H&E). A blinded evaluation by a pathologist was performed for all biopsy samples and using the muscle injury score [22–24]. For the muscle samples at end of recovery phase, a mean score was calculated for comparison.

### Statistical analysis

Continuous data are reported as median and error with range. Perfusion parameters and histology score differences between groups were analyzed using 2-way ANOVA with multiple comparisons or using a mixed-effect analysis when necessary. Outliers were identified using ROUT, Q = 1%. All statistical analyses were performed using Prism 9 for Mac OSX (GraphPad Software, La Jolla, CA). p-Values less than 0.05 were considered to be significant.

## RESULTS

VCA procurement time of was less than 20 minutes and warm ischemic time (WIT) was less than 12 minutes in all 23 hindlimbs. The overall mean initial weight was 14.47 +/− 1.99 g.

### Loading phase

**Figure 3:**
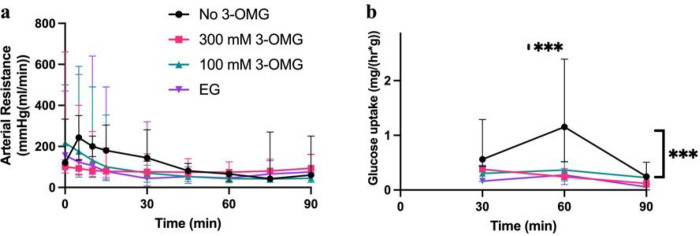
Perfusion parameters during loading phase. (**a**) Arterial resistance decreased from a mean of 206 +/− 167 mmHg(min/mL) at 5 min to 57 +/− 24 mmHg(min/mL) at 60 min as a sign of stabilization. While no significant difference if found, an increasing trend of the resistance can be observed as temperature is reduced from 21 to 4°C. (**b**) Glucose consumption per gram of tissue is significantly lower at 60 min in the no 3-OMG group (p≤ .0007), suggesting the VCAs being loaded with 3-OMG lose their ability to consume glucose, as intended. As temperature is reduced, glucose consumption also decreases in the no 3-OMG group due to suppression of the metabolism (p = .0003). *EG ethylene glycol; 3-OMG 3-O-Methyl-d-Glucose*

At 60 minutes maximum flow was reached in all groups, with a mean of .72 +/− .27 mL/min and no significant differences between groups. At 90 min flow was reduced as temperature was decreased to 4°C to .48 +/− .34 mL/min. As a result of stabilization early in the loading phase, arterial resistance decreased from a mean of 206 +/− 167 mmHg(min/mL) at 5 min to 57 +/− 24 mmHg(min/mL) at 60 min. Due to the decrease in flow towards the end of perfusion arterial resistance remained stable with a mean of 87 +/− 56 mmHg(min/mL) until the end of the loading phase in all groups, showing no significant differences between groups (Fig. 3).

All groups perfused with 3-OMG showed significantly lower glucose uptake at 60 min (p ≤ .0007). By the end of the loading phase as temperature and flow are reduced, the no 3-OMG group showed a clear decrease in glucose consumption (p = .0003).

Lactate release decreased in all groups over the course of perfusion from a mean of 5.7 +/− 2.0 to 2.8 +/− 1.1 mmol/L with only the 300 mM group showing a significant decrease between 30 and 90 min (p ≤ .0001). In contrast, potassium levels remain stable in all groups, with only an increase between 60 and 90 min in the 100 mM group (from 4.5 +/− .6 mmol/L to 5.9 +/− 1.3 mmol/L) as temperature was decreased to 4°C at the end of loading phase (p = .0204). Oxygen consumption was highest at 60 min in all groups, with the no 3-OMG group showing the largest fluctuations, reaching significance between 30 and 60 min (p = .0349).

Weight in the no 3-OMG group was stable between the start and the end of the loading phase, prior to the CPA flush (between − 2.99% and + .88% weight change). After CPA flush all VCAs in the no 3-OMG group lost between 15.93–22.9% weight. VCAs loaded with 3-OMG lost between 8.65–15.20% weight after 3-OMG loading and a total of 12.74–22.64% after CPA flush compared to initial weight.

### Recovery phase

**Figure 4:**
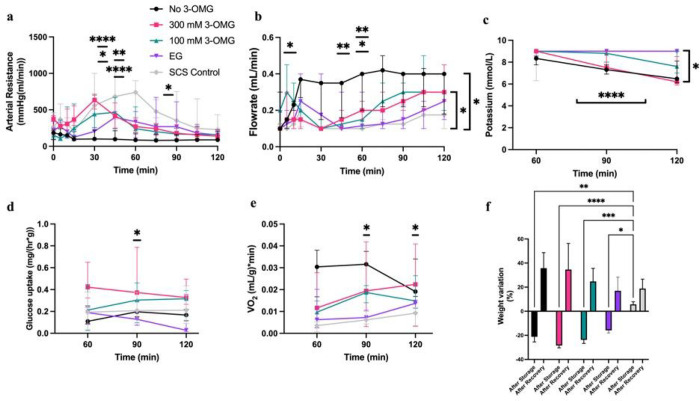
Perfusion parameters during recovery phase. (**a**) Arterial resistance decreases significantly faster in the supercooled groups. (**b**) Accordingly, flowrates increased faster in supercooled groups. (**c**) Potassium showed faster decrease in the no and 300 mM 3-OMG groups, showing correlation to faster increases in flowrates. (**d**) Glucose consumption restored to similar levels to SCS control group during recovery phase. (**e**) Oxygen consumption showed higher levels in supercooled groups early in the recovery phase. (**f**) Significant weight loss was found after 3-OMG loading and CPA flush in supercooled group. Weight gain during recovery phase seemed to show correlation to total weight gain at the end of recovery phase. * p≤.05 ** p≤.01*** p≤.001 **** p≤.0001

All limbs were successfully supercooled for 24h meaning VCAs and surrounding solution remained in the liquid phase while being at −4°C until the end of storage. Figure 4 shows the perfusion parameters during the recovery phase. Supercooled groups showed significantly faster decreases in arterial resistance during recovery phase with a mean of 272.7 +/− 132.1 mmHg(min/mL), while SCS control showed a mean of 717.5 +/− 164.2 mmHg(min/mL) at 60 min (p < .0001 for no 3-OMG and 300 mM; p = .002 for 100 mM; p = .0037 for EG compared to SCS control). In the no 3-OMG group this difference was most pronounced, showing significance from 45 until 115 min (p < .0001 and p = .019 resp.). Accordingly, flow could be increased earlier in recovery phase for the supercooled groups, especially when comparing no 3-OMG flow of .33 +/− .09 mL/min with .1 mL/min in SCS control group at 60 min (p = .0077).

Lactate release showed a decreasing trend in all groups over the duration of perfusion, with no significant differences between groups. Potassium release showed lower levels in the no 3-OMG group compared to EG (p = .0167) and to SCS control (p = .0149) at 120 min. Similarly, at 120 min 300 mM group showed lower potassium levels compared to EG (p = .001) and SCS control (p = .0003). Glucose consumption showed similar trends between groups. Oxygen consumption was higher in no 3-OMG group compared to SCS control at 60 (p = .0131) and 90 min (p = .042) recovering to comparable levels towards the end of perfusion.

During storage limited weight loss occurs in all supercooled groups, whilst in SCS control group limited weight gain occurs (data not shown).

Weight at the end of the storage phase is significantly lower in the supercooled groups compared to SCS control suggesting successful dehydration by the CPAs (Fig. 4F). However, while at end of study no 3-OMG and 300 mM group show highest and the EG and SCS control group the lowest weight gain and weight fluctuation, no significant differences were found.

### Histology

**Figure 5:**
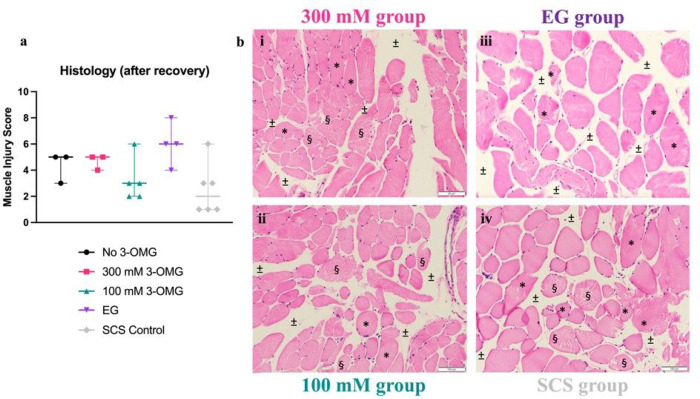
Histology of muscle samples after recovery. (**a**) Blinded, microscopic muscle injury score [22] shows no significant differences between groups, however EG group seems to have the highest scoring trend, and 100 mM group the lowest. (**b**) Light Microscopy (LM), x100, Hematoxylin and Eosin (H&E) staining shows myocyte size variation (*), myocyte damage (§) and interstitial edema (±), especially iii. EG showing major edema.

Microscopic histological analysis at the end of recovery phase is shown in Fig. 5. Lowest muscle injury scores are seen in 100 mM and SCS control group, although SCS control group showed the largest range between replicates. Contrary to weight gain, as mentioned earlier, EG group showed highest muscle injury scores. However, no significant differences between groups were found.

## DISCUSSION

This study demonstrates the development of a robust 24h storage protocol of an animal VCA model in a supercooled, ice-free state. Using machine perfusion VCAs were loaded with intracellular and extracellular CPAs before storage and recovered after storage resulting in a total preservation time of about 28h. Achieving a supercooled state in a complex model containing multiple tissue types and known ice nucleators such as hair, bone and nails poses challenges not encountered in solid organ supercooling. Having overcome these challenges, this study provides the starting point for developing VCA supercooling techniques that can extend storage for at least 48h enabling clinical application.

Earlier successes using these techniques were found in liver, kidney, lung, and heart models. Neonatal rodent kidneys were stored at −2°C for 48h in HTK, showing superior histological results to storage at 4°C for 24h [25]. In a rodent lung transplantation model, 17h storage at −2°C was compared to 4°C storage and fresh controls [26]. Supercooled lungs showed endothelial lining and perfusion parameters (tidal volume, oxygen levels, arterial pressure) and ATP levels that were comparable to fresh controls during the 60 min of reperfusion. In mice hearts, 96h storage at −8°C was achieved in a transplantation model and showed increased survival compared to storage at 4°C [27]. As a result of decreased myocyte metabolism, reduced ischemia-reperfusion injury (IRI), oxidative stress, and apoptosis of myocardial cells was seen. While this does not pertain to muscle function, this result is of special interest as it pertains to muscle injury – the most challenging tissue in a VCA in terms of preservation. In rodent livers, 100% transplantation survival after 3 days of storage, and 69% success after 4 days of storage was shown using supercooling techniques [16, 17]. Translating to human livers, 26h preservation was shown in a blood reperfusion model, more than doubling current preservation time in clinic [18]. As VCAs pose additional challenges, such as the presence of ice nucleating factors within the graft, multiple tissue types and an endothelial barrier with tight junctions, many variables necessitated adjustment to achieve a robust protocol.

In accordance with previous literature [16–18], the decrease in glucose consumption during the loading phase at 60 min suggests that 3-OMG loading requires between 30–60 min to observe its effects and suggest that metabolism is successfully depressed. One limitation here is that biochemical analysis may be impacted by the measurement of glucose as well as 3-OMG. However, the observation is supported by increasing oxygen consumption during recovery phase, which is not observed in the no 3-OMG group. Furthermore, the similar glucose consumption in all groups suggest that 3-OMG is successfully washed-out during recovery phase, allowing for glucose consumption. Another major observation is the weight loss of the VCAs during 3-OMG loading and CPA loading. As consequence, the no 3-OMG VCAs rapidly lose about one-fifth of their weight during CPA flushing alone. While the effect on total weight gain at the end of recovery phase does not seem to be influenced, it is known that quick weight changes can cause osmotic shock and be detrimental to cell survival [28, 29]. *In vitro* studies have investigated cell shrinkage following death, which has been found to be 25–28% of the original volume [30]. While the cell shrinkage observed in this supercooling protocol arises from osmotic changes and use of intra- and extracellular CPAs, these findings offer a valuable point of reference for considering the potential effects of cellular volume reduction. When comparing the different 3-OMG groups, it is notable that no 3-OMG achieves a higher flow rate earlier and does not show an increase in arterial resistance during recovery. Lactate and potassium levels are comparable to 300 mM group, however glucose consumption in the 100 and 300 mM 3-OMG is higher than the no 3-OMG group. Nonetheless, in terms of weight gain and histology score 100 mM group performs better than no 3-OMG and 300 mM group.

Another observation is that there seems to be a correlation in weight fluctuation between the weight loss at the end of storage and the total weight gain at the end of recovery phase. Using recovery solutions with higher osmolarities could be considered to reduce edema caused by abrupt changes in osmolarity as well as agents promoting membrane stability during cold storage. Furthermore, it is striking that EG group shows lowest weight gain and weight fluctuations while histology scores seem to be worse than the glycerol counterparts. Specifically in the edema scores, as well as the perfusion parameters such as the continuously high potassium levels and low oxygen and glucose consumption during recovery phase. A hypothesis could be an increased toxic effect on the cells by EG, inducing cell lysis, while its lower viscosity limits turbulence and thereby endothelial damage induced edema. It has been suggested that weight gain is an early indicator of graft dysfunction in VCAs [31, 32]. Therefore, the weight gain at the end of recovery should be of concern. However, the vasculature in rodent hindlimbs is small and could limit maintenance of adequate vascular pressure and thereby causing high hydrostatic pressures and endothelial damage due to shear stress, especially when perfusing at lower temperatures. Translation to large animal models would provide advantages such as larger vasculature, allowing for better control and adjustment to vascular resistance changes, limiting damage caused by machine perfusion, and increasing clinical relevance.

Despite achieving a substantial milestone and showing benefits of supercooling on organ health, weight fluctuations and edema remain a challenge in VCAs. As edema is determined by multiple factors explained by the revised Starling equation, the endothelium is a key contributor [33]. Membrane permeability is known to differ between organs as described by Staverman’s reflection coefficient. Furthermore, intercellular connections and permeability differ per organ. Moreover, skeletal muscle contains higher than usual numbers of capillaries. Other contributors are osmotic differences and vascular pressures. The exact causes of edema in the context of VCA preservation are yet to be elucidated, yet offer opportunities for therapeutic treatment and providing key solutions. Cryopreservation as a field is undergoing exciting developments with potential clinical applications. After successes in rodent and human liver supercooling, Tessier et al. showed successful 5-day storage of rodent livers using a technique called partial freezing, inspired by the rena sylvatica [19]. By using 3-OMG and CPA cocktails, ice formation occurs only in a slushy texture, thereby avoiding damaging, pointy ice crystals, and is containing this ice-like formation to the large vasculature. This technique enables storage up to −25°C, thereby reducing organ metabolism even further. More recently, Han et al. showed successful transplantation of 100-day vitrified rodent kidneys [34, 35]. By rapidly cooling to −150°C a glass-like state is achieved, enabling theoretically unlimited storage of organs. Beside the need for higher concentrations of toxic CPAs [36], the downside of this technique is the need for magnetic particles throughout the organ, and more importantly the need for expensive coils limited in size to enable rewarming without ice nucleation or cracking of the glass-like formation. While supercooling is not as stable, nor as durable, it does carry the potential to enable the necessitated two-day storage, whilst putting minimal stress on the organs without requiring extensive equipment. Furthermore, it allows for organ optimization by efficiently leveraging each of its phases. Machine perfusion during loading and recovery phase can be used for organ optimization, for administration of cell or gene therapies and for quality control determining transplantability, while storage can be utilized to increase exposure times of these therapies thereby increasing the versatility of this technique.

Future work should focus on achieving transplantable grafts [21]. SNMP provides a platform to establish such a protocol, which is especially important considering the multitude of variables rendering it prone to intersubjective variability. Machine perfusion allows for evaluation of preservation techniques and graft quality before requiring the use of recipient animals. Once metabolically acceptable results are achieved, next steps can be taken using transplantation models. Optimization could include enhancing loading of the VCAs with CPAs as such that cellular integrity is maintained and cells are protected from IRI as well as ice formation. This could extend to the use of agents protecting against IRI and suppressing cell death mechanisms, reducing toxicity of CPAs, or use of additional techniques such as isochoric freezing. Moreover, efforts should aim to investigate mechanistic causes of and solutions to edema in VCAs, such as the effect on the endothelial barrier, of changes in cellular volume and of the composition of the cytoplasm and interstitium. Additionally, functional assessment of muscle is lacking and would enable clinically relevant translation. Further optimization is expected to allow for the application of supercooling techniques in a transplantation model, which would be a breakthrough in the field of VCA preservation and open the doors to a wide range of applications. Developing technologies such as supercooling holds immense promise in extending the window of ischemic tolerance, thereby revolutionizing organ transplantation. The ability to prolong the time organs can survive outside the body is a game-changer, especially when considering the constraints imposed by travel time. This innovation has the potential to substantially increase the donor pool by mitigating the time sensitivity associated with organ transportation. Furthermore, in the context of allotransplantation for composite tissue grafts, where a myriad of parameters such as sex, HLA compatibility, anatomy, and skin color must align, the importance of an extended ischemic timeframe becomes even more pronounced. Supercooling technology emerges as a critical tool in overcoming logistical challenges, paving the way for more successful and nuanced transplant procedures.

This study demonstrates the practical feasibility of extending VCA preservation duration through supercooling in a rodent model and that the challenges of subzero non-freezing in ice-prone tissue can be overcome. A protocol that allows for reliable subzero non-freezing was established by orchestrating a combination of organ preservation solution and CPAs which are loaded and unloaded, with a deliberate emphasis on gradual temperature transitions. While the precise role of the use of 3-OMG in VCAs is nuanced and multifaceted, observations in this study suggest that lower concentrations (100 mM) may confer valuable advantages by facilitating a more gradual dehydration process. Furthermore, the use of ethylene glycol compared to glycerol as a main CPA shows benefits in the form of lower viscosity and improved weight change outcomes while metabolic parameters and histology suggest an increase in toxicity. In conclusion, this study contributes to knowledge of the limits of VCA preservation and can be used as a basis for further optimization to allow for transplantation following extended storage. The trajectory of future research and optimization within this realm holds promise for pushing the boundaries of VCA preservation, with profound implications for patients awaiting transformative transplantations.

## Data Availability

All data generated and analyzed during this study have been included in this manuscript and its Supplementary Information file unless stated otherwise. All raw data can be provided on request by the corresponding author.
